# A Swarm Optimization Solver Based on Ferroelectric Spiking Neural Networks

**DOI:** 10.3389/fnins.2019.00855

**Published:** 2019-08-13

**Authors:** Yan Fang, Zheng Wang, Jorge Gomez, Suman Datta, Asif I. Khan, Arijit Raychowdhury

**Affiliations:** ^1^School of Electrical and Computer Engineering, Georgia Institute of Technology, Atlanta, GA, United States; ^2^Department of Electrical Engineering, University of Notre Dame, Notre Dame, IN, United States

**Keywords:** ferroelectric FET, neuromorphic computing, spiking neural network, swarm intelligence, optimization

## Abstract

As computational models inspired by the biological neural system, spiking neural networks (SNN) continue to demonstrate great potential in the landscape of artificial intelligence, particularly in tasks such as recognition, inference, and learning. While SNN focuses on achieving high-level intelligence of individual creatures, Swarm Intelligence (SI) is another type of bio-inspired models that mimic the collective intelligence of biological swarms, i.e., bird flocks, fish school and ant colonies. SI algorithms provide efficient and practical solutions to many difficult optimization problems through multi-agent metaheuristic search. Bridging these two distinct subfields of artificial intelligence has the potential to harness collective behavior and learning ability of biological systems. In this work, we explore the feasibility of connecting these two models by implementing a generalized SI model on SNN. In the proposed computing paradigm, we use SNNs to represent agents in the swarm and encode problem solutions with the spike firing rate and with spike timing. The coupled neurons communicate and modulate each other's action potentials through event-driven spikes and synchronize their dynamics around the states of optimal solutions. We demonstrate that such an SI-SNN model is capable of efficiently solving optimization problems, such as parameter optimization of continuous functions and a ubiquitous combinatorial optimization problem, namely, the traveling salesman problem with near-optimal solutions. Furthermore, we demonstrate an efficient implementation of such neural dynamics on an emerging hardware platform, namely ferroelectric field-effect transistor (FeFET) based spiking neurons. Such an emerging *in-silico* neuron is composed of a compact 1T-1FeFET structure with both excitatory and inhibitory inputs. We show that the designed neuromorphic system can serve as an optimization solver with high-performance and high energy-efficiency.

## Introduction

Recent advances of deep learning models have initiated a resurgence of neural networks in the field of artificial intelligence (LeCun et al., 2015). Spiking Neural Network (SNN), as the third generation of neural networks, models the dynamic behavior of the biological neural system and focuses on the timing of the spikes (Maass, 1997). SNN utilizes spike timing to encode information and is capable of processing a significant amount of spatial-temporal information with a small number of neurons and spikes (Ghosh-Dastidar and Adeli, 2009; Ponulak and Kasinski, 2011). Meanwhile, neuromorphic computing hardware that implements SNN continue to gain increasing attention both in the industry and academia (Merolla et al., 2014; Davies et al., 2018). Moreover, recent progress of emerging nanotechnologies in devices and materials, such as resistive RAMs (RRAM) (Indiveri et al., 2013), spintronic devices (Romera et al., 2018) and metal-insulator transition (MIT) materials (Parihar et al., 2018), are facilitating real-time large-scale mixed-signal neuromorphic computing systems with the potential to bridge the energy efficiency gap between engineered systems and biological systems. SNN has been successfully applied in various computational tasks, such as visual recognition (Cao et al., 2015), natural language processing (Diehl et al., 2016), brain-computer interface (Kasabov, 2014), robot control (Bouganis and Shanahan, 2010). Recently, researchers have demonstrated ways to use networks of SNNs and similar neuromorphic systems to solve computationally more difficult problems. Of particular interest are optimization problems including NP-hard problem, such as constraint satisfaction problems (CSP) (Mostafa et al., 2015; Fonseca Guerra and Furber, 2017), vortex coloring problems (Parihar et al., 2017) and traveling salesman problems (TSP) (Jonke et al., 2016). These neural-inspired computing systems are designed exclusively so that the system converges at problem solutions by harvesting both deterministic as well as stochastic dynamics. Nonetheless, there are very few previous works about SNN based computing systems that address generic optimization problems. Although solving CSP with SNN is promising, it is enticing to note that the computational platform that we empirically find in the human brain can also solve complex optimization problems.

On the other hand, swarms of creatures also show collective behavior and evolve with complex and highly optimized global strategies. For example, a colony of ants is capable of planning the shortest path between their nest and their food sources, which is attributed to the collaborative deposit of chemical pheromone on the trails (Goss et al., 1989). A school of sardine naturally optimizes the movement of the swarm to minimize the loss when it is attacked by sharks (Norris and Schilt, 1988). Bees can build hives with an optimized structure in spatial efficiency and locate nearest nectar source plants with temporal efficiency (Michener, 1969). These swarms are composed of individuals that have inferior intelligence and simple behaviors. However, they exhibit highly intelligent collective behavior resulting from the collaboration. Inspired from these natural swarms, Swarm Intelligence (SI) constructs the computational models that describe the collaborative behaviors in decentralized and self-organized systems (Blum and Li, 2008). In recent years, SI is also applied to a wide range of fields, such as path planning, control of robotics, image processing, and communication networks (Duan and Luo, 2015). Examples of classic SI optimization methods include ant colony optimization (ACO) (Dorigo and Di Caro, 1999), particle swarm optimization (PSO) (Kennedy and Eberhart, 1999). More advanced SI optimization algorithms that have been proposed recently include the firefly algorithm (FA) (Fister et al., 2013) and bat algorithm (Yang, 2010).

SNN and SI are apparently two computational intelligence models that differ in concepts, architectures and applications. SNN is inspired by the neural system of a high-intelligent individual, while SI mimics the collaborative behavior of somewhat simpler creatures. However, these two sets of models share some similarities. Both of them are bio-inspired, highly parallelized, and composed of multiple homogeneous units (agents and neurons) (Fang and Dickerson, 2017). Their computational capabilities origin from the interaction and communication between the individual units. For example, both of the neurons in SNN and agents in SI exhibit the behavior of phase and frequency synchronization. From the perspective of computational neuroscience, synchronization of oscillatory neural activity is currently one of the attractive areas of research, due to its close connection to the rhythms of the brain, seizures in epileptic patients and tremor in Parkinson patients (Guevara Erra et al., 2017). Neural synchronization has also been utilized in neuromorphic computing based on spiking or oscillatory neural networks, such as visual processing (Fang et al., 2014), olfactory processing (Brody and Hopfield, 2003), and solving constraint satisfaction problems (Parihar et al., 2017). In these applications, neural synchronization usually indicates the completeness of computing and the stable state of dynamical systems that presents the results. Similarly, an SI model can be viewed as a discrete dynamical system with an energy function that matches the objective function of the optimization problem. Agents perform collaborative searches and eventually synchronize and cluster around the global energy minima, which represents the global optimal (or near-optimal) solution. Such synchronization phenomena in SNN and SI model are the primary inspiration of our work.

As the problem dimension and the swam sizes increase, SI algorithms can become computationally expensive in terms of delay and power. On the other hand, SNNs cannot harness the collective properties of optimization problems. In our previous work (Fang and Dickerson, 2017), we explored the opportunities in bridging these two models and proposed a computing paradigm based on SI and coupled spiking oscillator network to address optimization problems. In this work, we provide details and develop an SI-SNN architecture and demonstrate how it is capable of solving two types of optimization problems, parameter optimization of continuous objective functions and TSP.

Along with algorithm development, the next generation of computing systems must harness the computational advantages of emerging post-silicon technologies. In particular, for neuromorphic systems, research has started in earnest to identify materials and device systems that exhibit the inherent dynamics of bio-inspired neurons and synapses. Various competing technologies are being explored, including insulator-metal-transition devices (Parihar et al., 2017), RRAMs (Ielmini, 2018), spintronic neurons and synapses (Romera et al., 2018) as well as scaled silicon CMOS implementations (Indiveri and Horiuchi, 2011). In this paper, we explore the use of ferroelectric field-effect transistor (FeFET) based spiking neurons in the design of the proposed SI-SNN architecture. An algorithm-hardware co-design is required to provide the next breakthrough in computational efficiency, in particularly for neuro-inspired systems whose dynamics can be simulated, albeit inefficiently in a von-Neumann system. The FeFET based spiking neuron is a compact 1T-1FeFET *in-silico* neuron with both excitatory and inhibitory inputs (Wang et al., 2017). It takes advantage of the hysteresis of the FeFET and operates as a relaxation oscillator that periodically generates voltage spikes. We extract a simplified model to capture the critical voltages and spike timing of FeFET based spiking neuron. This compact model enables the simulation of SNN that contains a large number of neurons.

First, we show how the proposed SI-SNN organizes multiple SNNs and performs parallel meta-heuristic searching, which is conducted by a swarm of collaborative agents in an SI-inspired algorithm. In this design, the spiking neurons encode the parameters of the agents with the spiking rate, interact with each other via spikes and search for globally optimal solutions. The agents that find better solutions modulate the firing rates of neurons in other agents. The modulation behavior is performed through event-based synaptic connections. Specifically, the excitatory input voltage of a post-synaptic FeFET neuron is modulated by a small amount whenever a spike arrives. Eventually, the optimal solution is represented by the firing rates when the entire swarm synchronizes.

In the second problem demonstration, we use a similar SI-SNN computing architecture to imitate the ACO (Dorigo and Di Caro, 1999) algorithm and show how it is capable of solving the TSP. Each SNN is a winner-takes-all (WTA) network and the order of its neurons' spikes represents the traveled route (solution candidate) of a single agent (ant). The synaptic weight is updated online by the spikes and shared by multiple SNNs, resembling the pheromone trails in ACO. The travel routes of SNNs are adapted according to the distances between cities and the pheromone distribution. Consequently, the optimal solution eventually evolves though such a parallel search process.

The remaining sections of this paper are organized as follow. In Materials and Methods, we describe the dynamical behavior model of FeFET spiking neuron as a hardware platform; it is the neuron model we use to develop the SI-SNN computing paradigm. Then we introduce two SI-SNN paradigms and demonstrate solutions to different optimization problems—continuous objective functions and TSP. In section Results, we provide the simulation results of our proposed method. In the final section, we draw conclusions.

## Materials and Methods

### Neuromorphic Hardware Technology

Owing to the continuous dynamics of the biological nervous systems biomimetic SNNs are much less efficient when they are executed on digital computing machines. Neuromorphic hardware that specifically supports SNN has been explored theoretically and experimentally for three decades (Mead, 1989). Nowadays neuromorphic engineering focuses on developing large-scale neural processing systems for cognitive tasks (Indiveri et al., 2011). In this work, we demonstrated a co-design of the proposed SI-SNN computing paradigm and neuromorphic hardware, where the hardware natively implements the required neuronal dynamics. A neuromorphic hardware system, comprises of two fundamental functional units:

- *Neuron*: This is the primary focus of this paper. Here, we explore the spiking dynamics of a FeFET neuron based on its excitatory and inhibitory interfaces and utilize this dynamical behavior to enable different SNN functionalities. The FeFET neuron has also been proven to be energy-efficient. It costs about one-third of power as traditional CMOS circuits and can potentially achieve the energy efficiency of 0.36 nJ/spike with 45 nm FinFET process (Wang et al., 2018). We discuss the detail dynamical behavior of FeFET spiking neuron in the next section.
- *Synapse*: Various resistive memory technologies are currently being investigated to realize synaptic behavior. The synapse does not show complex dynamics, but rather allows summation of the outputs of multiple pre-synaptic neurons to modulate the membrane potential of the post-synaptic neuron. For the sake of brevity, we do not include a detailed discussion about the hardware implementation of synapses because many emerging device technologies can fulfill the requirements of SI-SNN systems (Kuzum et al., 2013).

### Ferroelectric Based Spiking Neuron

**Figure 1:**
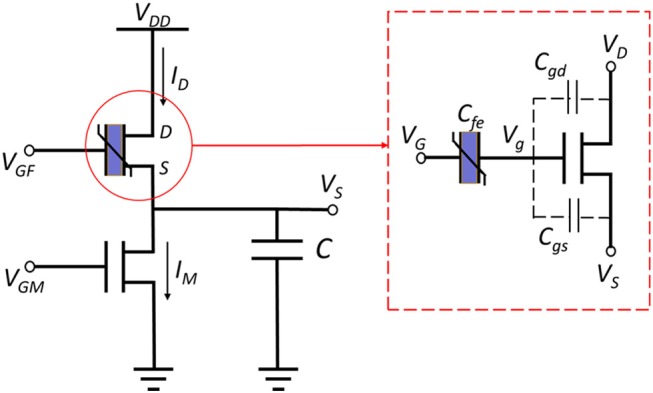
FeFET based spiking oscillator consists of a FeFET and a normal NMOS transistor that are used to charge and discharge a capacitor. The FeFET (red box) can be view as a ferroelectric layer that connected to a common FET (3D model of FeFET is shown in Figure S1).

**Figure 2:**
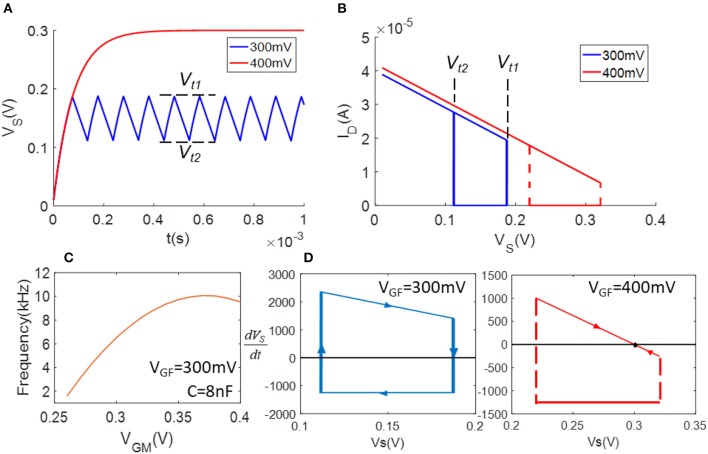
Demonstration of model simulation: **(A)** waveforms of *V*_*S*_ (*V*_*GF*_ = 300 mV and 400 mV); **(B)** *I*_*D*_ – *V*_*S*_ plot shows the hysteresis loops of *I*_*D*_ in **(A)**; **(C)** *V*_*GM*_ v.s. frequency as *V*_*GF*_ = 300 mV; **(D)** flow diagrams of equation.

FeFET is a semiconductor device that has a similar structure as the MOSFET or FinFET, except that an additional layer of ferroelectric (FE) material is integrated into the stack of gate terminal (Aziz et al., 2018). The spontaneous polarization of the FE layer is reversible under a certain electric field applied in the correct direction. The polarization depends on the current electric field and its history, resulted in a hysteresis loop. For further details, interested readers are pointed to Aziz et al. (2018). Such a feature of FE layer induces a FeFET to switch “on” at a high voltage and “off” at a low applied gate voltage. Figure 1 illustrate the structure of a FeFET (red box). A relaxation oscillator based on FeFET was recently proposed in Wang et al. (2017). Furthermore, the proposed oscillator was utilized to implement a spiking neuron with excitatory and inhibitory interfaces (Wang et al., 2018). The proposed circuits employ the hysteresis of a FeFET and a traditional NMOS transistor to periodically charge and discharge a load capacitor and generate spikes of voltage (Figures 1, 2A). Figure S1 shows a 3D view of the FeFET and the NMOS transistor.

The FeFET based neuron has only two transistors and exhibits an advantage in the energy efficiency of spikes, which is discussed later in section Results. More importantly, this neuron model is capable of modeling multiple neural dynamics that has been observed in cortical and thalamic neurons. We can use two gate voltages, V_GM_ and V_GF_, of two transistors to imitate the excitatory and inhibitory synaptic inputs, respectively of biological neurons, and thus enable various neural firing patterns (Fang et al., 2019). In this section, we describe a compact behavior model of the FeFET based spiking neuron. This model captures the critical switching voltages of FeFET and computes the current that controls spike timing (phase) and spiking frequency. It neglects the complex physical transitions before device switching and reduces the computing cost tremendously, enabling the simulation of large scale SNN built on FeFET neuron.

Figure 1 depicts the schematic of a FeFET spiking neuron (Wang et al., 2017). It is a relaxation oscillator that charges and discharges the load capacitor repetitively with *I*_*D*_ and *I*_*M*_, which are the currents flowing through the FeFET and the NMOSFET. The former one injects current to capacitor *C* and the latter one provides a discharging path. To briefly explain the oscillation, we assume *V*_*GF*_, *V*_*GM*_, and *V*_*DD*_ are all fixed. If we start from the charging phase, the potential across the capacitor, *V*_*S*_, is low and thus the *V*_*GS*_ of FeFET is large enough to set the FE layer to coercion and inject charge into the gate node *V*_*g*_ and quickly switches on the FeFET. As a result, *I*_*D*_ increases rapidly and charges the capacitor until the end of this phase. As the capacitor gets charged and *V*_*S*_ rises, the discharging phase begins. The FE layer reaches the opposite coercive threshold, drains the charge from *V*_*g*_ and switches the FeFET to an OFF state. In this phase, *I*_*D*_ is very small and *I*_*M*_ gets a chance to discharge the capacitor. Due to the decrease of *V*_*S*_ again, the whole cycle repeats with these two phases. Therefore, *V*_*S*_ keeps swinging between the two critical voltages *V*_*t*1_ and *V*_*t*2_. In Figure 2A, the blue waveform plots the trace of *V*_*S*_, illustrates the Fast Spiking mode of a spiking neuron.

#### Dynamic Behavior Model

Because the switching process of FeFET is fast when compared to the oscillation period, we assume the switching of FeFET is instant in our model. We are primarily interested in the timing of the spike, instead of other physical metrics of the FeFET device. We focus our model on the critical voltages when FeFET switches and the current that charges and discharges the capacitors. Details of the model have been presented elsewhere (Fang et al., 2019) and we summarize the key findings here for the sake of completion. It is also important to point out the key neuronal dynamics that are achievable in the FeFET neuron, that can be harnessed in the SI-SNN computational framework. Critical voltages *V*_*t*1_ and *V*_*t*2_ depend on the properties of FeFET, *V*_*G*_ and *V*_*D*_ (*V*_*GF*_ and *V*_*DD*_) fed into the gate and drain terminals. To capture *V*_*t*1_ and *V*_*t*2_, we only need to aim at the boundary conditions when the FeFET switches. Thus, we can write the equation based on charge (Fang et al., 2019):

(1) $$\begin{array}{l} V_{g}C_{T}=Q_{fe}+C_{fe}V_{GF}+C_{gd}V_{DD}+C_{gs}V_{S}\\ \;C_{T}=C_{fe}+C_{gd}+C_{gs} \end{array}$$

where, *Q*_*fe*_ is the released bond charge. Here *V*_*g*_ = *V*_*GF*_ – *V*_*fe*_. *V*_*fe*_ is the potential across the FE layer and equals to one of the two coercive voltages, *V*_*c*1_ and *V*_*c*2_. Therefore, we can compute the critical voltages of switching, *V*_*t*1_ and *V*_*t*2_ as (Fang et al., 2019):

(2) $$\begin{array}{r} {V_{t}}_{i}=\alpha^{\left(i\right)}-\gamma^{\left(i\right)}V_{DD}+\left(1+\gamma^{\left(i\right)}\right)\left(V_{GF}-V_{ci}\right),i=1,2\\ \gamma^{\left(i\right)}=\frac{{C_{gd}}^{\left(i\right)}}{{C_{gs}}^{\left(i\right)}},\alpha^{\left(i\right)}=-\left(\frac{C_{T}{V_{c}}^{\left(i\right)}+\beta^{\left(i\right)}Q_{fe}}{{C_{gs}}^{\left(i\right)}}\right),\beta^{\left(1,2\right)}=\pm 1 \end{array}$$

*i* = 1,2 represent the cases of switching on and off. α^(i)^, γ^(i)^, *V*_*c*1_, and *V*_*c*2_are device parameters that can be calibrated via experimental measurements (Wang et al., 2018) or estimated from physics-based models. Thus, we can obtain *V*_*t*1_ and *V*_*t*2_ in terms of *V*_*GF*_ and *V*_*DD*_. An alternative method to obtain *V*_*t*1_ and *V*_*t*2_ is to calibrate the data experimentally from circuits. In the case we shown here, we have (*V*_*t*1_ = 187 mV, *V*_*t*2_ = 111 mV) when *V*_*GF*_ = 300 mV (*V*_*t*1_ = 320 mV, *V*_*t*2_ = 219 mV), when *V*_*GF*_ = 400 mV.

With *V*_*t*1_ and *V*_*t*2_, we can model the dynamical behavior of the FeFET based neuron with a first-order non-linear differential equation for *V*_*S*_:

(3) $$\begin{array}{l} \frac{dV_{S}}{dt}=\frac{1}{C}\left(sI_{D}-I_{M}\right),\{\begin{array}{l} s=0,V_{t1}\to V_{t2}\\ s=1,V_{t1}\leftarrow V_{t2} \end{array}\\ \;I_{D}=g_{F}(V_{g}-V_{S}-V_{Gth})\\ \;I_{M}=g_{M}(V_{GM}-V_{Mth}) \end{array}$$

In Equation (3), we use a binary variable *s* to set the current in two phases. When *s* = 1, the load capacitor is being charged, while *s* = 0 represent the discharging phase. *I*_*D*_ and *I*_*M*_ are modeled with two piecewise linear functions. Transistor parameters *g*_*F*_*, g*_*M*_, *V*_*Gth*_, and *V*_*Mth*_ are transconductances and threshold voltages. *V*_*g*_ is calculated from Equation (1).

Compare to physics-based FeFET models proposed in previous works (Aziz et al., 2016; Lenarczyk and Luisier, 2016), our model is more concise and friendly to the system-level simulation of SNN. Despite the simplicity, we still need to capture the timing of spikes accurately. We verify the model by utilizing it to recreate the dynamic behaviors and data provided in Wang et al. (2017). In this case, we adopt the same configuration and parameters in Wang et al. (2017), in which the FeFET is a 14 nm FinFET node that connects to a 10 nm HfO_2_ FE layer with mode detail description in Khandelwal et al. (2017). The NMOS transistor is a FinFET but without the FE layer. For the circuits simulation, we use the default settings of *V*_*DD*_ = 400 mV, *V*_*GM*_ = 350 mV and *C* = 8 nF. Here we use *g*_*F*_ = *g*_*M*_ = 10^−4^S, *V*_*Mth*_ = 250 *mV*, and *V*_*g*_ − *V*_*Gth*_ ≈ 400*mV*.

We simulate the circuits with varying values of *V*_*GF*_ and *V*_*GM*_ and demonstrate the results in Figure 2. Figure 2A plots two waveforms of *V*_*S*_ when *V*_*GF*_ = 300 mV and *V*_*GF*_ = 400 mV. It is worth noting that when *V*_*GF*_ = 300 mV, the hysteresis of FeFET produces normal oscillation; when *V*_*GF*_ = 400 mV, *V*_*S*_ operates between a higher range of *V*_*t*1_ and *V*_*t*2_, which leads to a balance between the charging and discharging of capacitors and cease the oscillation. Figure 2B draws the *I*_*D*_ – *V*_*S*_ curves of each case, showing the FeFET's hysteretic behavior under *V*_*GF*_ = 300 mV. To explain the condition of oscillation, Figure 2D plots the flow diagram of the FeFET based oscillator. When *V*_*GF*_ = 300 mV, the x-axis *dV*_*S*_/*dt* = 0 intersects the steep transition of the hysteretic loop. As a result, there is no attractor or fixed point but a limit cycle in the system to generate oscillations. On the other hand, when *V*_*GF*_ = 400 mV, the first derivative of *V*_*S*_ passes the charging phase of the hysteretic loop and forms a fixed point near *V*_*S*_ = 300 mV. The fixed point creates a stable state that eliminates the oscillation. Let us assume *V*_*S*_ as the membrane voltage of a neuron, its non-oscillatory state can be viewed as the resting state. The FeFET based oscillator exhibits similar dynamics as a LIF neuron, except that it fires spikes with an opposite direction. Namely, the FeFET spiking neuron fires when *V*_*S*_ reaches the low threshold voltage, *V*_*t*2_, and the action potential of spikes is reversely integrated from *V*_*DD*_ to 0. Such a dynamical behavior is validated experimentally in Wang et al. (2018) (Figures S3, S4). If we fix *V*_*GF*_, *V*_*GM*_ can be used to tuning the firing rate of the FeFET spiking neuron. The *V*_*GM*_ and frequency curve showed in Figure 2C here is measured as the instantaneous firing rate of spikes, instead of the mean frequency obtained from the power spectrum.

In summary, high *V*_*GF*_ suppress the spiking activities of the FeFET neuron and keep it at the resting state, thus exhibiting a prototypical “inhibitory” behavior. When the inhibition of *V*_*GF*_ is disabled, raising *V*_*GM*_ increases the firing rate, and the corresponding input behaves as an “excitatory” interface.

#### Biomimetic Neuronal Dynamics

The traditional Leaky Integrate-and-Fire (LIF) Neuron model is not able to cover the dynamics of multiple ion channels of biological neurons due to its simplicity of one dimension. Izhikevich (2003) proposed a 2-D neuron model that efficiently reproduces various dynamics of cortical neurons. The innovation of Izhikevich's model is to use a slow variable to control the leak current of a LIF model. Inspired from such a design, we propose to take advantage of inhibitory input *V*_*GF*_ in FeFET spiking neuron to imitate the function of the “slow variable” because the FeFET is responsible for the “resetting” phase (discharging) of a spike (Fang et al., 2019). Associated with the frequency adaption enabled by excitatory input *V*_*GM*_, our neuron model can imitate multiple types of firing patterns (Fang et al., 2019). We demonstrate two types of spiking dynamics that we utilize for SNN based computation for this work. These two types of firing patterns are respectively:

- FS and LTS (Fast Spiking and Low-Threshold Spiking): firing patterns found in inhibitory cortical cells. They both feature with spike trains in high frequency. LTS has a frequency adaptation. We treat them as one firing pattern (FS) for the simplicity of representation in proposed computing paradigms.
- RS (Regular Spiking): a regular cortical firing pattern with relatively low-frequency.

**Figure 3:**
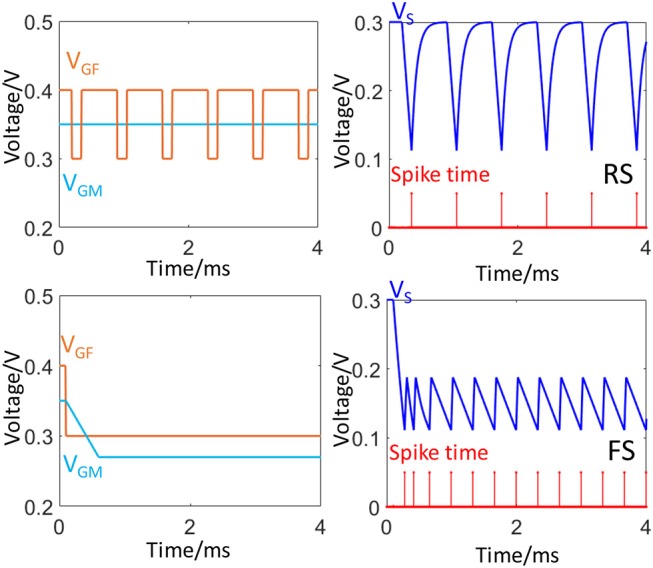
Two neural firing patterns, FS and RS. The plots on the left column show the waveforms of input signals to *V*_*GF*_ and *V*_*GM*_. The right column plot the waveforms of *V*_*S*_ (blue) and corresponding timing of spike train (red).

Figure 3 illustrates how the application of different configuration of *V*_*GF*_ and *V*_*GM*_ can generate these two firing patterns. Besides FS and RS, the FeFET spike neuron model is also capable of imitated other firing patterns such as Intrinsically Busting (IB), Chattering (CH), and interested readers are pointed to Fang et al. (2019) for further discussions. In the FS mode, the FeFET neuron operates in an oscillatory mode with disabled inhibition (low *V*_*GF*_) for a high frequency of firing. Meanwhile, *V*_*GM*_ can be used to adjust the firing frequency. In RS mode, spikes are generated through a periodic inhibitory input which has a large duty cycle. In the original design of FeFET spiking neuron (Wang et al., 2018), the polarity of the spike train is inverted using an output inverter and the input gate voltages, *V*_*GF*_ and *V*_*GM*_ accept voltage spikes from pre-synaptic neurons via RC integrators. The two spiking modes, FS, and RS can be set by using proper input of spiking trains. Figure S2 illustrates the frequency modulation via spikes.

### Swarm Intelligence (SI)—Spiking Neural Network (SNN) Optimization

Having established the electronic equivalent of the biological neuron, we now focus on the algorithm development which can harness the dynamics of this neural circuit. In this section, we introduce the SI-SNNs that imitates the collective behavior of SI algorithms. First, we provide a general framework of SI algorithms. Then, we describe the architectures of two SI-SNNs, which are aimed at two different optimization problems, respectively.

#### SI Algorithm Framework

To define the problem, we use the general form of optimization, which is to find a solution of *x* to maximize/minimize the objective/cost function *f*(*x*) under certain constraints. Namely, *x* = argmin*f*(*x*), s.t constraint. For the parameter optimization of continuous objective functions, we do not take constraints into consideration.

Different SI algorithms are distinct from each other due to the different swarm behaviors they mimic. However, a general framework can be developed to fit most of these algorithmic principles. In the beginning, a swarm is initialized with multiple “agents.” Each agent's location coordinates in the solution space represent the parameters of the solution. In each iteration of the optimization process, the agents move and search for solutions by updating their parameters. Such a collaboration operation is meta-heuristic and trades off between the randomization and the performance of the local search. To locate the optimal solution and to escape from local minima simultaneously, each agent follows particular behavioral rules and seek to balance exploration and exploitation (Crepinsek et al., 2011). Exploration determines the swarm's capability of discovering new candidates of the global solution. On the contrary, exploitation focuses on the individual local search within the vicinities of the current best solution. The pseudo-code in Algorithm 1 describes the framework of most SI algorithms (Fang and Dickerson, 2017).

**Algorithm 1:** General SI Frameworks

1: **Initialize** swarm *S* with *m* agents {*s*1, *s*2, …, *sm*}
2: **While** *t* < *MAX*_*ITER* or condition satisfy **do**
3: Update vector of parameters $s_{i}^{t}=s_{i}^{t-1}+\Delta s_{i}^{t-1}$ for each
$s_{i}^{t-1}\in S^{t-1}$
4: Evaluate $f\left(s_{i}^{t}\right)$ for each $s_{i}^{t}$
5: Compute each $\Delta s_{i}^{t}$ for the next iteration based on $f\left(s_{i}^{t}\right)$
6: *t* = *t*+1
7: **end while**

Each agent *si* in swarm *S* is an *n*-dimension vector that represents the variable of *f*(*x*) ∈ ℝ^n^ → ℝ. The behavior rule of agent that compute $\Delta s_{i}^{t}$ vary among different SI algorithms. For example, PSO updates *si* based on the history of both the best global and local solutions. FA only requires the current global best solution. Despite this distinction, SI algorithms are flexible and model-free because of their similar characteristics in meta-heuristic search. In other words, the same method can be used to address different types of optimization problems.

#### SI-SNN Model Architecture for Continuous Objective Function

**Figure 4:**
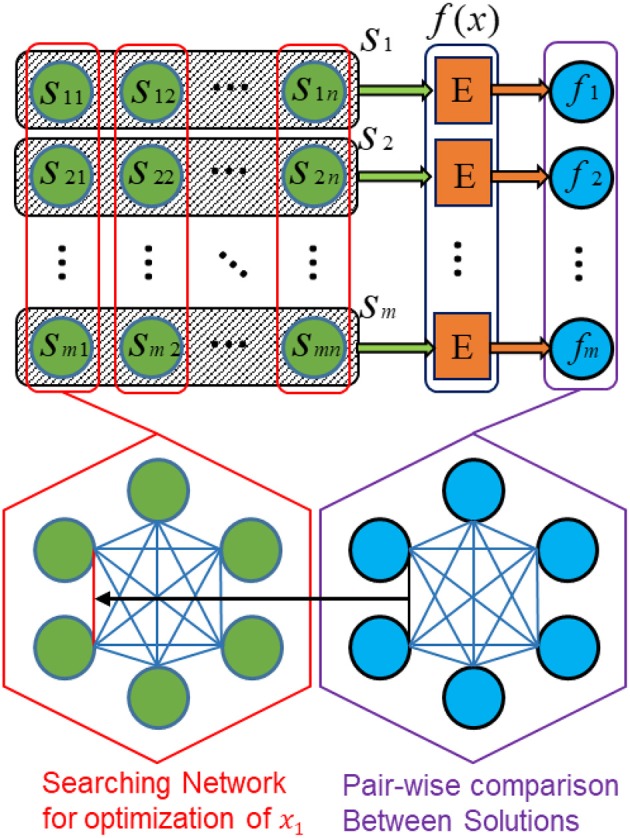
SI-SNN architecture for parameter optimization of continuous functions.

Figure 4 depicts the architecture of the proposed SI-SNN for optimizing the parameters of continuous objective functions. Following the configuration and notation as Algorithm 1, we consider a swarm of *m* agents for an *n*-dimension problem. Accordingly, we prepare an *m* × *n* array of neurons (labeled as green) to represent a parameter *s*_*ij*_ (1 < *i* < *m*, 1 < *j* < *n*) in each agent *s*_*i*_. The black frame with shadow encloses the neurons that belong to the **agent** *s*_*i*_. The red frame indicates the neurons that compose the **searching network** for the optimization of one parameter *x*_*k*_ (1 < *k* < *n*). Namely, each **column** of neurons is a fully connected spiking neural network defined as a searching network. Each **row** of neurons represents an agent. The block **E** (labeled as orange) evaluates the solution found by each agent by computing the value of the objective function *f*(*x*). The computing platform of block *E* depends on the different optimization tasks and objective functions. For compatibility, it can be another spiking neural network (Iannella and Back, 2001), or a digital/mixed-signal computing hardware, or feedback from the external environment gathered through sensors such in reinforcement learning problems. The evaluation of each solution found by an individual agent produces an *m*-sized column vector (labeled as blue). These solutions are compared to each other and used to guide the synaptic update of the neurons.

In section Ferroelectric Based Spiking Neuron, we introduced the FeFET spiking neuron and several of its biomimetic patterns. In this scenario, we explore the use of frequency (firing rate) of each neuron to represent the value of a parameter. Therefore, an adaptable voltage-controlled high-frequency spiking mode is necessary. We choose the **FS** mode of FeFET spiking neuron (Figure 3), in which the inhibitory input is off (*V*_*GF*_ = 300 mV) and the voltage of the capacitor *V*_*S*_ oscillates between *V*_*t*1_ = 111 mV and *V*_*t*2_ = 188 mV. The firing rate is tuned by the excitatory input, *V*_*GM*_ (Figure 2C).

In a searching network, each neuron belongs to a different agent. Its firing rate represents the value of the specific parameter in the current solution. The firing rates are initialized by setting *V*_*GM*_ with random values normally distributed in a specific range. During the optimization process, these neurons adjust each other's firing rates based on the results of the pairwise comparison between solutions, following the rule described in Equation (4). For the *i*th neuron in a searching network, we have

(4) $$V_{GMi}=V_{GMi}+\Delta v_{ij}+\theta \eta ,\text{ on spike from }{\text{j}}^{\text{th}}\text{ neuron}$$

$$\Delta vij=\{\begin{array}{l} w(V_{GMj}-V_{GMi}),if\;f(s_{i})<f(s_{j})\\ 0,\;otherwise \end{array}$$

where η is a Gaussian noise term and θ is a scaling factor of the stochastic term. Equation (4) explains an event-based rule of updating *V*_*GM*_. Once a spike from the pre-synaptic neuron *j* arrives at the post-synaptic neuron *i* and if the *j*th agent has a better solution than the *i*th agent, *V*_*GMi*_ is updated by adding the difference between *V*_*GMi*_ and *V*_*GMj*_ so that it becomes more close to *V*_*GMj*_, which reduces the difference between the firing rates of the two neurons. *w* is the synaptic weight that controls the step size of the *V*_*GM*_ modulation. This synaptic rule is applied to all the neurons and enables the agents with better solutions to dominate other agents by tuning their firing rate. But the dominant agents change behavior as the searching process continues. Sometimes passive agents may find better solutions as a result of a stochastic search and become active and start to modulate the neurons of other agents. The searching process ends when the neurons in every searching networks are synchronized with near-identical frequencies. Such a swarm behavior is inspired by fireflies, which attract each other via the frequency synchronization of their flash signaling (Fister et al., 2013).

#### SI-SNN for Traveling Salesman Problem

TSP is an NP-hard combinatorial optimization problem. Given the distance between nodes in a graph, the goal of TSP is to find a path that visits all the nodes in the graph exactly once with minimal total distance. Among SI algorithm family, ant colony optimization algorithm (ACO) was proposed to solve TSP (Dorigo and Di Caro, 1999). ACO is a swarm-based method inspired by the collaborative behavior of ants. Different from the rest of the SI algorithms, the agents (ants) in ACO do not send information to each other directly but leave the shared information (pheromone) on the edge of graphs (Dorigo and Di Caro, 1999). Individual ant makes decisions based on the concentration of pheromone on their travel route. We define a trip as complete when an agent finishes visiting all the nodes. In a trip, the amount of pheromone on the edge is updated by all the ants that have passed by that edge and further influence their choice of route in the next trip. An iteration is defined as an event when all the agents have finished one trip. After a certain number of iterations, the best route eventually converges to the optimal solution.

Before we design the SI-SNN for ACO, we notice that a fully connected SNN with *n* neurons can be mapped onto a graph of *n*-city TSP (Hopfield and Tank, 1985) and the travel route can be indicated by the order of spikes (Jonke et al., 2016). However, the behavior of a swarm of ants is difficult to be represented simultaneously by the spike train within a single SNN. Therefore, we use multiple SNNs to simulate the trip of each ant. For each SNN, the difficulty in the design of dynamics lies on how to make each neuron fire only once and follow the correct order in one trip. In previous work (Jonke et al., 2016), multiple WTA SNNs are used to show the travel path of one trip. By exerting the inhibitory and excitatory interfaces of FeFET spiking neurons, we can use the spike train of a single SNN to represent the travel path of one agent.

**Figure 5:**
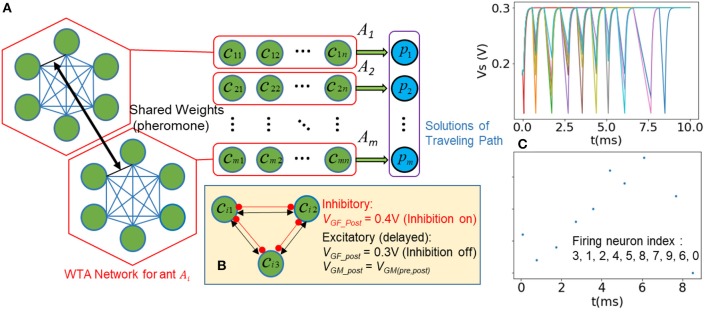
**(A)** Modified SI-SNN architecture for solving TSP **(B)** inhibitory and excitatory synapses enable that only one neuron fires after latest pre-synaptic spike in a WTA network **(C)** Plot of state variable Vs and the raster plot that shows the order of fired spikes in one trip.

Figure 5A shows the modified architecture of SI-SNN for solving TSP. We start with an *m* × *n* array of neurons (green) and each neuron represents a city (node) *c*_*ij*_ (1 < *i* < *m*, 1 < *j* < *n*) in the travel path of the agent (ant) *A*_*i*_. A red frame indicates a fully-connected WTA network, which models the traveling behavior of an ant *A*_*i*_. In one trip, each neuron in a WTA network only fires once and the solution of the TSP *p*_*i*_ (labeled as blue) is represented as the order of firing of a spike train. The collaboration between agents does not rely on the evaluation of *p*_*i*_. Hence, the SI-SNN architecture for ACO has no feedback loop and search networks as shown in the previous section. Instead, these WTA networks simultaneously access and update a set of shared weights that mimic the pheromone trails of the ant colony. Meanwhile, to enable the winner-takes-all mechanism, we employ an instant inhibitory synapse and a delayed excitatory synapse to pair-wise connect every neuron in the WTA network. Accordingly, we use the regular spiking (RS) mode of FeFET neuron. Namely, after the inhibition input *V*_*GF*_ was set to low, the capacitor of FeFET neuron needs to be discharged from the resting state 300 mV to the threshold voltage 111 mV to generate a spike. We describe the dynamical behavior of one WTA network (Figure 5B) as follow:

Step 1. The weight of pheromone τ_*ij*_ between any neuron *i* and *j* is initialized as 1. The inhibition of neuron is disabled (*V*_*GF*_ = 300 mV). A randomly selected neuron is set as the start node with *V*_*GM*_ = 350 mV and the rest neurons are initialized with *V*_*GM*_ < 350 mV.
Step 2. The neuron of the starting node generates the first spike before the rest of the neurons reach the firing threshold and immediately set their inhibition to a high state through the inhibitory synapse, defined as (*V*_*GF*_*post*_ = 400 mV on a pre-synaptic spike). In such a circumstance, all the neurons instantly switch to the charging stage. After they reach the resting state at 300 mV, the fired neuron will be set as inhibited till the end of the current trip, while the rest of the neurons are triggered by the delayed excitatory synapse, which is defined as:
  (5) $$\{\begin{array}{l} V_{GF\_post}=300mV\\ V_{GM\_post}=\kappa \frac{{\tau_{ij}}^{p}}{{D_{ij}}^{q}}+\theta \eta +V_{Mth}\;(\text{after delay }\Delta \text{t}\\ \text{ on pre-synaptic spike}) \end{array}$$
where the *i* and *j* are indices of pre-synaptic and postsynaptic neurons, *D*_*ij*_ is the distance between two nodes. *p* and *q* are the weights of the pheromone and the distance between the nodes, used for balancing the global and local information. κ and θ are scaling factors and η is the Gaussian random term. The rest of the neurons, which have not fired any spike yet, are free from inhibition and start to discharge (integration stage). However, their discharge rate is controlled by the *V*_*GM*_*-*, depending on the amount of pheromone, τ_*ij*_ and *D*_*ij*_ in Equation (5).
Step 3. The neuron that discharges the fastest become the winner, fire the second spike of this trip and inhibit other neurons. The shared weight of pheromone between the two neurons that fires in a sequence is updated as:
  (6) $$\begin{array}{r} \tau_{ij}=\left(1-\rho \right)\tau_{ij}+\frac{\omega }{D_{ij}mn} \end{array}$$
where ρ is a decay factor, which represents the vaporization of pheromone and encourages agents to explore new routes. ω is the scaling factor of the increasing amount of pheromone.
Step 4. The whole process (Step 1 ~3) is repeated until all the neurons in the WTA network fire a spike.
To demonstrate this process clearly, we plot the trace of *V*_*S*_ of neurons and the raster plot of a WTA network in Figure 5C. The raster plot indicates the firing order of spikes in a trip of a 10-city TSP (solution provided in **Figure 8**).
During the optimization, the process described above is executed by *m* WTA networks simultaneously and the pheromone trails are shared and updated on the fly. Once all the WTA networks (agents) complete a trip, a new iteration starts with the updated pheromone weights. The whole optimization process terminates when the maximum iteration number is reached.

## Results

### Parameter Optimization of Continuous Functions

We simulate the SI-SNN computing paradigm with BRIAN, an open source SNN simulator based on Python (Stimberg et al., 2014). We use the dynamical model discussed in Section 2.2 to simulate FeFET based spiking neurons. For the first demonstration, the continuous objective function we aim at is the 2-D Schwefel's function:

(7) $$\begin{array}{r} f\left(x\right)=\overset{n}{\underset{i=1}{\sum }}\sin\left(\sqrt{\left|x_{i}\right|}\right) \end{array}$$

**Figure 6:**
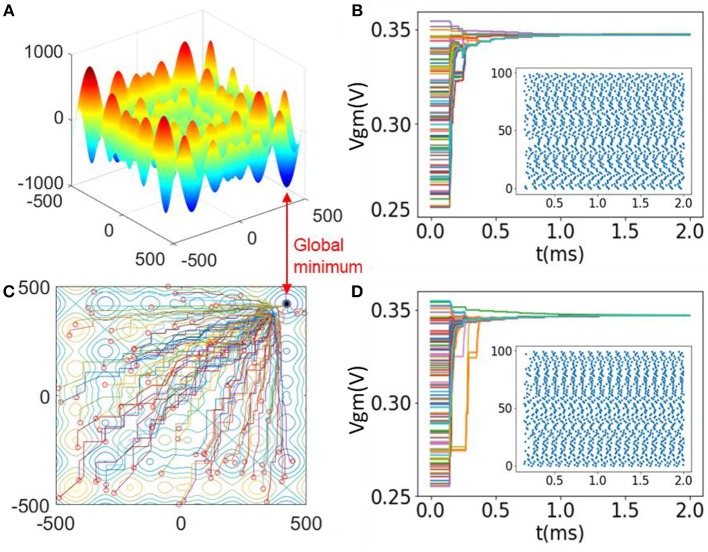
**(A)** Landscape of 2-D Schwefel's function; **(B)** Contour map with solution traces of each agent in the optimization process of **(A)**; **(C,D)** Evolution of *V*_*GM*_ in two searching networks with raster plots.

The dimension of this function is *n* = 2, and *x*_*i*_ ∈ [−500, 500]. This function has more than 50 local minima and a global minimum at ***x*** = (418.92, 418.92). Figure 6A plots the landscape of 2-D Schwefel's function as a 3-D surface. In this case, we prepare an SI-SNN with 100 agents and two searching networks (*m* = 100, *n* = 2). The scaling factor of random noise θ = 0.02. For such a configuration, we randomly initialize the *V*_*GM*_ of each FeFET spiking neuron in the range of [255 mV, 355 mV] with a uniform distribution. Consequently, the firing rates of neurons range from 0.801 to 9.852 kHz in **FS** mode and are mapped to the range of *x*_*i*_ ∈ [−500, 500]. We note that when the network synchronizes, the *V*_*GM*_ of most of the neurons cluster around 339 mV and the firing rates are stabilized at 9.186 kHz. Such a value of *V*_*GM*_ corresponds to the global minima where *x*_*i*_ = 418.92. There exist errors between the parameter represented by the firing rate due to the nonlinearity in the *V*_*GM*_ - Frequency curve. It needs to be calibrated and compensated in the hardware design. In this simulation, we did not consider a hardware implementation of the evaluation blocks. Figures 2C,D plots the *V*_*GM*_ of each neuron in two searching networks along the optimization process. The convergence of the SI-SNN takes 1.5 ms, which is ~14 cycles of spiking. Meanwhile, we notice that the firing rates of a few of the neurons are initially attracted to local minima and then get pulled out by the neurons of other agents with better solutions. This phenomenon indicates that SI-SNN model is capable of escaping from the “trap” of local minima. Figures 6C,D also show the raster plots of all the spikes during the simulation process. Figure 6B is a contour map of Figure 6A with the traces of the best solutions found by each agent during the optimization. The red circles mark the initial positions of 100 agents in the solution space. Eventually the swarm converges into the global minimum.

**Figure 7:**
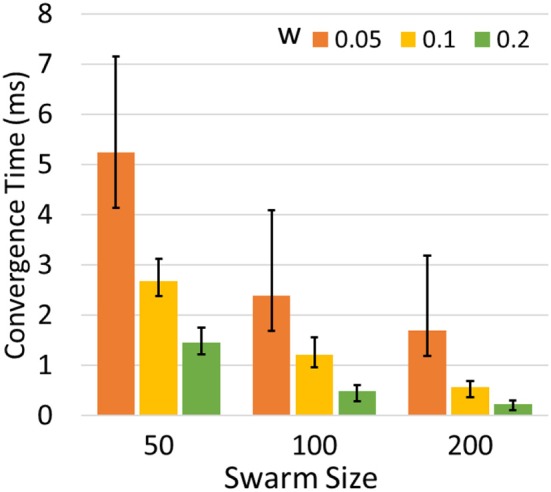
Average convergence time to optimize 2-D Schwefel's function in different m and w. The error bars indicate the maximum and minimum time cost.

We set synaptic weight *w* and swarm size *m* to different values and run the simulation 200 times for each configuration. Figure 7 shows the average time for the optimization problem under different configurations of w and *m*. The result indicates that larger *m* and *w* can speed up the optimization process. However, the best choice of *w* falls within a certain range. An extremely large or small value may lead to failure in synchronization or the network may miss of global optimum. Having more agents improves the efficiency and performance of optimization but also increases the demands for computing resources.

Apart from Schwefel's function, we also test the SI-SNN on several other benchmark objective functions with different dimensions. The equations and landscape of these benchmark functions can be found in Pohlheim (2005). For the evaluation of the optimization performance, we use Relative Percentage Deviation (RPD), which we defined as the absolute percentage error between the objective function evaluation of best solution founded by algorithms and the correct optimal solution.

(8) $$\begin{array}{r} RPD=\frac{abs\left(f\left(best\right)-f\left(opt\right)\right)}{f\left(opt\right)}\times 100\% \end{array}$$

Table 1 show the average convergence time with corresponding standard deviation and the success rate in finding the near optima with an RPD smaller than 2%. In such a test, we employ swarms with 200-agent to optimize the parameter of four benchmark functions. In these simulations, we keep the same configuration of the FeFET neuron model. The time constants are the same as previous tests and the firing frequencies of neurons still range from 0.801 to 9.852 kHz. The parameters such as time and voltage, are scalable with different devices and capacitors in the FeFET based circuits, e.g., smaller capacitors may reduce the time of charge and discharge from microsecond to nanosecond (Wang et al., 2018).

**Table 1:** Parameter optimization of benchmark objective functions.

**Benchmark function (dimension)**	**Convergence time (Mean ± Std)**	**Success rate**
Michalewicz's (*n* = 16)	348 ± 98 ms	89%
Schwefel's (*n* = 64)	782 ± 223 ms	92%
Ackley's (*n* = 128)	1,379 ± 928 ms	99%
De Jong's (*n* = 256)	945 ± 105 ms	100%

### Solving TSP

**Figure 8:**
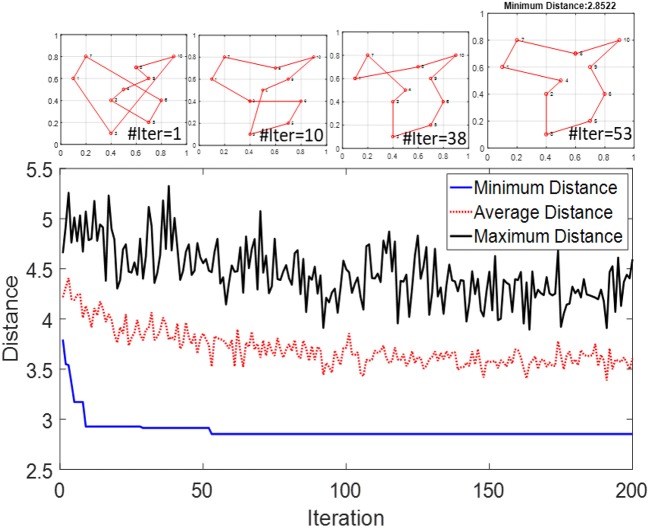
Distance of the best solution to a 10-city TSP in each iteration of SI-SNN.

We use the same method to simulate the modified SI-SNN model for solving TSP. However, since the simulator does not support conditionally terminating the simulation process, we run each iteration separately in sequence. After all the WTA networks finish the trip of their agents, we reset the system and continue to run the next iteration with the updated pheromone weights. Each iteration contains *m* × *n* spikes but the time cost only depends on how fast the slowest agent fires *n* spikes. The whole simulation process ends when the maximum iteration number is reached. The performance and convergence speed of the original ACO are sensitive to the hyperparameters. In the simulations of this section, we set the swarm size twice as the size of the problem (*m* = 2*n*), κ = 0.01, θ = 0.03, ρ = 0.03, ω = 2. For *q* and *p*, it is recommended to use values within 2 and 4. However, to reduce the complexity of the hardware design, we can set both of them to 1. Figure 8 demonstrates the optimization process of solving a 10-city TSP. It demonstrates the distances of solutions searched in each iteration and display the best route in several iterations. The optimal travel route was found at the 53rd iteration.

Next, we run a set of benchmark tests with our customized 10-city TSP and four other TSP from a standard TSP library TSPLIB. The sizes of these problems are respectively [10,16,29, 48, 52]. For each problem, we run the optimization 200 times using SI-SNN and also using SNNs that performs random-walk-based searches without any shared information (pheromone). Table 2 shows the mean and standard deviation of iteration numbers to reach the best solution and the corresponding RPD. The standard deviation is not shown for multi-SNN random search because the successful runs are fewer than five times and such a strategy fail to find any near-optimal solution when the problem size increases. The results in Table 2 demonstrate that without collaboration, the random search performed by a swarm is much less effective. We also notice that for complex TSPs, the SI-SNN can only approach near-optimal solutions due to the limitations inherited from the original ACO algorithm.

**Table 2:** Performance of solving TSP.

**TSP benchmark problem**	**Iteration number (Mean ± Std) (SI-SNN/Multi-SNN random Walk)**	**Performance (RPD %)**
my10 (*n* = 10)	59 ± 28/343	0
ulysses16 (*n* = 16)	147 ± 75/1,751	0
bays29 (*n* = 29)	364 ± 143/Fail	0
att48 (*n* = 48)	625 ± 237/Fail	3%
berlin52 (*n* = 52)	574 ± 126/Fail	1%

In Table 3, we estimate the “time taken” and “energy consumption” of several methods that implement ACO to solve a 48-city TSP. Bali et al. (2016) provides the performance of ACO executed respectively by a GPU and a CPU on laptop, although the 48-city TSP they use may not be att48. We conservatively estimate the energy cost of GPU and CPU based on their idle power consumption, and subtract the power consumed by the onboard memory. For the SI-SNN, we compared the time and energy cost between FeFET spiking neuron and a few of the previous literature on silicon-based neurons. We calculate the estimation results with the total spike numbers, timing, and energy cost per spike. In this scenario, we do not consider the delay and power consumption of synapses and assume the neurons of previous works is also compatible with the WTA network in SI-SNN. For FeFET based spiking neurons, we provide two sets of data, 45 nm FinFET process with *C* = 8 nF and 14 nm FinFET process with *C* = 1 pF. The first one has a relatively lower frequency in the kHz range and higher energy consumption of ~0.36 nJ/spike. The second one uses a predictive transistor technology and a smaller capacitor that generates oscillation frequency in the MHz range. The comparison in Table 3 shows that the FeFET based SI-SNN is a promising computing paradigm for optimization in terms of high performance and energy efficiency. Even with traditional CMOS, event-based SI-SNN is highly energy efficient compared to CMOS digital systems. Compared with silicon neurons, we observe that post-CMOS, emerging devices can effectively reduce the number of transistors as well by harnessing the inherent neuronal dynamics. In particular, the FeFET spiking neuron provides both excitatory and inhibitory interfaces, which benefits the design of the WTA network. It reduces the number of neurons and synapses. For example, without inhibition input directly to the neuron, representing one trip of N-city TSP requires *N* × *N* neuron (Jonke et al., 2016), while we only use a single N-neuron WTA network in this work. Thus, the energy reduction brought by the unique feature of FeFET spiking neuron is not shown in Table 3.

**Table 3:** Comparison of proposed computing paradigm and other methods.

**Task: ACO for 48-city TSP**	**SI-SNN**				**GPU**	**CPU**
**References**	**Indiveri (****2003****)**	**Wijekoon and Dudek (****2008****)**	**Babacan et al. (****2016****)**	**Wang et al. (****2017**, **2018****) used in this work**	**Bali et al. (****2016****)**	**Bali et al. (****2016****)**
Technology	CMOS analog	CMOS analog	Memristor+CMOS	FeFET+CMOS	CMOS digital	CMOS digital
Manufacturing process	1.5 μm	0.35 μm	0.18 μm	45 nm (14 nm)	40 nm	22 nm
Neuron model (Dynamics)	LIF (RS)	Izhkevich (RS, FS, CH, IB)	Izhkevich (RS, FS, CH, IB)	Izhkevich (RS, FS, CH, IB)	GPU model: GTX 480M	CPU model: Intel Core T i7-4700MQ
Device count/neuron (total)	18T (172.8k)	14T (134.4k)	1T+3M (38.4k)	1T+1FeFET (19.2k)	3 billion on chip	1.4 billion on chip
Synaptic input	Excitatory	Excitatory	Excitatory	**Excitatory+Inhibitory**	/	/
Time cost	5 min	36 ms	3 s	3.9 s (0.48 ms)	2.4 s	6.8 s
Energy consumption of neuron in total	90 mJ	0.1 mJ	0.5 mJ	2.2 mJ (~nJ)	~190J	~320J

## Discussion

In this paper we propose SI-SNN as a computational platform based on FeFET based spiking neurons. We observe that:

1. The FeFET based spiking neurons exhibit rich neuronal dynamics. In the SI-SNN architecture, we use the rate-based representation in the FS mode for the optimization of the continuous objective function and the phase-based representation in the RS mode for solving TSP. To the best of our knowledge this is one of the first demonstrations of a computing platform that harnesses various neuronal dynamics for solving different optimization problems.
2. The inhibitory input of FeFET spiking neuron facilitates the design of the WTA network in solving TSP. In our design, the spiking behavior of neurons can inhibit and compete with each other, and naturally mimic path planning of ants. Without the inhibitory interface, more hardware resources are required.
3. The design of FeFET spiking neuron is compact. The entire circuit can run at high frequency with low energy cost.
4. The dynamical behavior model we extract is simple and effective. It can capture the spike timing but bypass the complex physical equations of ferroelectric devices, and improve the efficiency of the simulation.

Given the simulation results of the first SI-SNN model in section Parameter Optimization of Continuous Functions, we observe two tradeoffs between the metrics of continuous function optimizations. The first one is between the spatial cost and the temporal cost. A larger size of a swarm results in faster speed of convergence but also requires more neurons and spike generators, which is equivalent to the tradeoff between efficiency and energy. The second one is between convergence speed and accuracy. A larger network weight and less randomization may improve the efficiency of the search process but also increases the risk of missing the optima. In particular, the random term in metaheuristic search becomes increasingly important as the problem dimension increases, because the search routine covers less of a solution space in a higher dimension. These observations can be used to tune model parameters.

In the SI-SNN TSP solver, our design benefits from the dynamical feature of FeFET based spiking neurons. The excitatory and inhibitory interfaces enable the design of the WTA embedded in each SNN. The simulation results emphasize the importance of shared information between agents in the collaborative search process of swarms. Further work can be pursued by invoking more ACO algorithms such as Max-min ant systems (MMAS) (Stützle and Hoos, 2000) and ant colony system (ACS) (Dorigo and Gambardella, 1997) that can improve the performance and convergence speed at the cost of more complicated hardware design.

As far as the hardware implementation is concerned, the solution-based adaption of synaptic parameters can be realized with address-event representation (AER) systems (Park et al., 2012) or memristor crossbar arrays (Long et al., 2016; Ielmini, 2018). The random terms in the synaptic rule can be implemented via the emerging stochastic devices such as spintronic device and memristors (Vincent et al., 2015). Furthermore, future works may harness more learning properties from synapse models with non-linear dynamics. Also, the interplay between swarm intelligence and individual cognitive intelligence is a research area that remains active (Rosenberg et al., 2016). The results will have contributions to fields as varied as multi-agent artificial intelligence, social psychology, cognitive science and so on.

In summary, we propose a new SNN computing paradigm built on FeFET spiking neuron that combines swarm intelligence in agents of spiking neural network to address optimization problems. We simulate our SI-SNN model with SNN simulator and demonstrate its capability to optimizing parameters of continuous objective functions and for solving the traveling salesman problem. In our design, we utilize two types of neural dynamics, FS and RS, to encode information with firing rate and spike timing, respectively, to perform varying computational tasks. The FeFET based SNN is a promising hardware platform for achieving the energy-efficiency and high-performance denoted by future computing systems (Wang et al., 2018). We demonstrate the computational power of neuromorphic systems in the field of general optimization problems. Above all, our work sheds light on the connection between individual intelligence and swarm intelligence.

## Data Availability

No datasets were generated or analyzed for this study.

## Author Contributions

YF proposed the method of SI-SNN and performed the simulation and data analysis. AR and YF formulate the problem and drafted the manuscript. JG, ZW, SD, and AK worked on the device and circuits of FeFET spiking neuron.

### Conflict of Interest Statement

The authors declare that the research was conducted in the absence of any commercial or financial relationships that could be construed as a potential conflict of interest.

## References

[B1] Aziz A. Breyer E. T. Chen A. Chen X. Datta S. Gupta S. K. (2018). Computing with ferroelectric FETs: devices, models, systems, and applications, in Proceedings of IEEE Design, Automation and Test in Europe Conference and Exhibition (DATE) (Washington, DC: IEEE).

[B2] Aziz A. Ghosh S. Datta S. Gupta S. K. (2016). Physics-based circuit-compatible SPICE model for ferroelectric transistors. IEEE Electron Device Lett. 37, 805–808. 10.1109/LED.2016.2558149

[B3] Babacan Y. Kaçar F. Gürkan K. (2016). A spiking and bursting neuron circuit based on memristor. Neurocomputing 203, 86–91. 10.1016/j.neucom.2016.03.060

[B4] Bali O. Elloumi W. Abraham A. Alimi A. M. (2016). ACO-PSO optimization for solving TSP problem with GPU acceleration, in International Conference on Intelligent Systems Design and Applications (Cham: Springer).

[B5] Blum C. Li X. (2008). Swarm intelligence in optimization, in Swarm Intelligence, eds Blum C. Merkle D. (Berlin, Heidelberg: Springer), 43–85.

[B6] Bouganis A. Shanahan M. (2010). Training a spiking neural network to control a 4-dof robotic arm based on spike timing-dependent plasticity, in The 2010 International Joint Conference on Neural Networks (IJCNN) (Washington, DC: IEEE).

[B7] Brody C. D. Hopfield J. J. (2003). Simple networks for spike-timing-based computation, with application to olfactory processing. Neuron 37, 843–852. 10.1016/S0896-6273(03)00120-X 12628174

[B8] Cao Y. Chen Y. Khosla D. (2015). Spiking deep convolutional neural networks for energy-efficient object recognition. Int. J. Computer Vis. 113, 54–66. 10.1007/s11263-014-0788-3

[B9] Crepinsek M. Mernik M. Liu S. H. (2011). Analysis of exploration and exploitation in evolutionary algorithms by ancestry trees. Int. J. Innovat. Comput. Appl. 3, 11–19. 10.1504/IJICA.2011.037947

[B10] Davies M. Srinivasa N. Lin T. H. Chinya G. Cao Y. Choday S. H. Liao Y. (2018). Loihi: a neuromorphic manycore processor with on-chip learning. IEEE Micro 38, 82–99. 10.1109/MM.2018.112130359

[B11] Diehl P. U. Zarrella G. Cassidy A. Pedroni B. U. Neftci E. (2016). Conversion of artificial recurrent neural networks to spiking neural networks for low-power neuromorphic hardware, in Proceedings of IEEE International Conference Rebooting Computing (ICRC) (Washington, DC: IEEE).

[B12] Dorigo M. Di Caro G. (1999). Ant colony optimization: a new meta-heuristic, in Proceedings of the 1999 Congress on Evolutionary Computation-CEC99 (Cat. No. 99TH8406) (Washington, DC: IEEE).

[B13] Dorigo M. Gambardella L. M. (1997). Ant colony system: a cooperative learning approach to the traveling salesman problem. IEEE Transac. Evol. Comput. 1, 53–66. 10.1109/4235.585892

[B14] Duan H. Luo Q. (2015). New progresses in swarm intelligence–based computation. Int. J. Bio-Inspired Comput. 7, 26–35. 10.1504/IJBIC.2015.067981

[B15] Fang Y. Dickerson S. J. (2017). Achieving swarm intelligence with spiking neural oscillators, in 2017 IEEE International Conference on Rebooting Computing (ICRC) (Washington, DC: IEEE).

[B16] Fang Y. Gomez J. Wang Z. Datta S. Khan A. I. Raychowdhury A. (2019). Neuro-mimetic dynamics of a ferroelectric FET based spiking neuron. IEEE Electron Device Lett. 40, 1213–1216. 10.1109/LED.2019.2914882

[B17] Fang Y. Yashin V. V. Seel A. J. Jennings B. Barnett R. Chiarulli D. M. (2014). Modeling oscillator arrays for video analytic applications, in Proceedings of IEEE/ACM International Conference on Computer-Aided Design (Washington, DC: IEEE).

[B18] Fister I. Fister I. Jr. Yang X. S. Brest J. (2013). A comprehensive review of firefly algorithms. Swarm Evol. Comput. 13, 34–46. 10.1016/j.swevo.2013.06.001

[B19] Fonseca Guerra G. A. Furber S. B. (2017). Using stochastic spiking neural networks on spinnaker to solve constraint satisfaction problems. Front. Neurosci. 11:714. 10.3389/fnins.2017.00714 29311791 PMC5742150

[B20] Ghosh-Dastidar S. Adeli H. (2009). Spiking neural networks. Int. J. Neural Syst. 19, 295–308. 10.1142/S0129065709002002 19731402

[B21] Goss S. Aron S. Deneubourg J. L. Pasteels J. M. (1989). Self-organized shortcuts in the Argentine ant. Naturwissenschaften 76, 579–581. 10.1007/BF00462870

[B22] Guevara Erra R. Perez Velazquez J. L. Rosenblum M. (2017). Neural synchronization from the perspective of non-linear dynamics. Front. Computat. Neurosci. 11:98. 10.3389/fncom.2017.00098 29123478 PMC5662639

[B23] Hopfield J. J. Tank D. W. (1985). Neural computation of decisions in optimization problems. Biol. Cybernet. 52, 141–152. 4027280 10.1007/BF00339943

[B24] Iannella N. Back A. D. (2001). A spiking neural network architecture for nonlinear function approximation. Neural Netw. 14, 933–939. 10.1016/S0893-6080(01)00080-6 11665783

[B25] Ielmini D. (2018). Brain-inspired computing with resistive switching memory (RRAM): devices, synapses and neural networks. Microelectronic Eng. 190, 44–53. 10.1016/j.mee.2018.01.009

[B26] Indiveri G. (2003). A low-power adaptive integrate-and-fire neuron circuit, in Proceedings of the 2003 IEEE International Symposium on Circuits and Systems, ISCAS'03 (Washington, DC: IEEE).

[B27] Indiveri G. Horiuchi T. K. (2011). Frontiers in neuromorphic engineering. Front. Neurosci. 5:118. 10.3389/fnins.2011.00118 22013408 PMC3189639

[B28] Indiveri G. Linares-Barranco B. Hamilton T. J. Van Schaik A. Etienne-Cummings R. Delbruck T.. (2011). Neuromorphic silicon neuron circuits. Front. Neurosci. 5:73. 10.3389/fnins.2011.00073 21747754 PMC3130465

[B29] Indiveri G. Linares-Barranco B. Legenstein R. Deligeorgis G. Prodromakis T. (2013). Integration of nanoscale memristor synapses in neuromorphic computing architectures. Nanotechnology 24:384010. 10.1088/0957-4484/24/38/384010 23999381

[B30] Izhikevich E. M. (2003). Simple model of spiking neurons. IEEE Transac. Neural Netw. 14, 1569–1572. 10.1109/TNN.2003.820440 18244602

[B31] Jonke Z. Habenschuss S. Maass W. (2016). Solving constraint satisfaction problems with networks of spiking neurons. Front. Neurosci. 10:118. 10.3389/fnins.2016.00118 27065785 PMC4811945

[B32] Kasabov N. K. (2014). NeuCube: a spiking neural network architecture for mapping, learning and understanding of spatio-temporal brain data. Neural Netw. 52, 62–76. 10.1016/j.neunet.2014.01.006 24508754

[B33] Kennedy J. Eberhart R. C. (1999). The particle swarm: social adaptation in information-processing systems, in New Ideas in Optimization, eds Corne D. Dorigo M. Dasgupta D. Moscato P. Poli R. Price K. V. (Maidenhead: McGraw-Hill Ltd.), 379–388.

[B34] Khandelwal S. Duarte J. P. Khan A. I. Salahuddin S. Hu C. (2017). Impact of parasitic capacitance and ferroelectric parameters on negative capacitance FinFET characteristics. IEEE Electron Device Lett. 38, 142–144. 10.1109/LED.2016.2628349

[B35] Kuzum D. Yu S. Wong H. P. (2013). Synaptic electronics: materials, devices and applications. Nanotechnology 24:382001. 10.1088/0957-4484/24/38/382001 23999572

[B36] LeCun Y. Bengio Y. Hinton G. (2015). Deep learning. Nature 521:436. 10.1038/nature14539 26017442

[B37] Lenarczyk P. Luisier M. (2016). Physical modeling of ferroelectric field-effect transistors in the negative capacitance regime, in IEEE International Conference on Simulation of Semiconductor Processes and Devices (SISPAD) (Washington, DC: IEEE).

[B38] Long Y. Jung E. M. Kung J. Mukhopadhyay S. (2016). Reram crossbar based recurrent neural network for human activity detection, in 2016 International Joint Conference on Neural Networks (IJCNN) (Washington, DC: IEEE).

[B39] Maass W. (1997). Networks of spiking neurons: the third generation of neural network models. Neural Netw. 10, 1659–1671. 10.1016/S0893-6080(97)00011-7

[B40] Mead C. A. (1989). Analog VLSI and Neural Systems. Reading, MA: Addison-Wesley.

[B41] Merolla P. A. Arthur J. V. Alvarez-Icaza R. Cassidy A. S. Sawada J. Akopyan F.. (2014). A million spiking-neuron integrated circuit with a scalable communication network and interface. Science 345, 668–673. 10.1126/science.1254642 25104385

[B42] Michener C. D. (1969). Comparative social behavior of bees. Ann. Rev. Entomol. 14, 299–342. 10.1146/annurev.en.14.010169.001503

[B43] Mostafa H. Müller L. K. Indiveri G. (2015). An event-based architecture for solving constraint satisfaction problems. Nat. Commun. 6:8941. 10.1038/ncomms9941 26642827 PMC4686837

[B44] Norris K. S. Schilt C. R. (1988). Cooperative societies in three-dimensional space: on the origins of aggregations, flocks, and schools, with special reference to dolphins and fish. Ethol. Sociobiol. 9, 149–179. 10.1016/0162-3095(88)90019-2

[B45] Parihar A. Jerry M. Datta S. Raychowdhury A. (2018). Stochastic IMT (insulator-metal-transition) neurons: an interplay of thermal and threshold noise at bifurcation. Front. Neurosci. 12:210. 10.3389/fnins.2018.00210 29670508 PMC5893757

[B46] Parihar A. Shukla N. Jerry M. Datta S. Raychowdhury A. (2017). Vertex coloring of graphs via phase dynamics of coupled oscillatory networks. Sci. Rep. 7:911. 10.1038/s41598-017-00825-1 28424457 PMC5430425

[B47] Park J. Yu T. Maier C. Joshi S. Cauwenberghs G. (2012). Live demonstration: hierarchical address-event routing architecture for reconfigurable large scale neuromorphic systems, in 2012 IEEE International Symposium on Circuits and Systems (Washington, DC: IEEE).

[B48] Pohlheim H. (2005). Geatbx examples examples of objective functions, in Documentation for GEATbx version 3.7 (Genetic and Evolutionary Algorithm Toolbox for use with Matlab) (Washington, DC: IEEE).

[B49] Ponulak F. Kasinski A. (2011). Introduction to spiking neural networks: information processing, learning and applications. Acta Neurobiol. Exp. 71, 409–433. Available online at: https://ane.pl/archive?vol=71&no=4&id=7146 22237491 10.55782/ane-2011-1862

[B50] Romera M. Talatchian P. Tsunegi S. Araujo F. A. Cros V. Bortolotti P.. (2018). Vowel recognition with four coupled spin-torque nano-oscillators. Nature 563:230. 10.1038/s41586-018-0632-y 30374193

[B51] Rosenberg L. Baltaxe D. Pescetelli N. (2016). Crowds vs swarms, a comparison of intelligence, in 2016 Swarm/Human Blended Intelligence Workshop (SHBI) (Washington, DC: IEEE).

[B52] Stimberg M. Goodman D. F. Benichoux V. Brette R. (2014). Equation-oriented specification of neural models for simulations. Front. Neuroinformatics 8:6. 10.3389/fninf.2014.00006 24550820 PMC3912318

[B53] Stützle T. Hoos H. H. (2000). MAX–MIN ant system. Future Generat. Computer Syst. 16, 889–914. 10.1016/S0167-739X(00)00043-1

[B54] Vincent A. F. Larroque J. Locatelli N. Romdhane N. B. Bichler O. Gamrat C.. (2015). Spin-transfer torque magnetic memory as a stochastic memristive synapse for neuromorphic systems. IEEE Transac. Biomed. Circuits Syst. 9, 166–174. 10.1109/TBCAS.2015.2414423 25879967

[B55] Wang Z. Crafton B. Gomez J. Xu R. Luo A. Krivokapic Z. (2018). Experimental demonstration of ferroelectric spiking neurons for unsupervised clustering, in 2018 IEEE International Electron Devices Meeting (IEDM) (Washington, DC: IEEE).

[B56] Wang Z. Khandelwal S. Khan A. I. (2017). Ferroelectric oscillators and their coupled networks. IEEE Electron Device Lett. 38, 1614–1617. 10.1109/LED.2017.2754138

[B57] Wijekoon J. H. Dudek P. (2008). Compact silicon neuron circuit with spiking and bursting behaviour. Neural Netw. 21, 524–534. 10.1016/j.neunet.2007.12.037 18262751

[B58] Yang X. S. (2010). A new metaheuristic bat-inspired algorithm, in Nature Inspired Cooperative Strategies for Optimization (NICSO 2010) (Berlin, Heidelberg: Springer).

