# Supplementary material for: A Swarm Optimization Solver Based on Ferroelectric Spiking Neural Networks
Source: Front Neurosci. 2019 Aug 13;13:855. doi: 10.3389/fnins.2019.00855 (PMC6700359; doi:10.3389/fnins.2019.00855)
Supplement: Supplementary file 1 [file Data_Sheet_1.PDF]

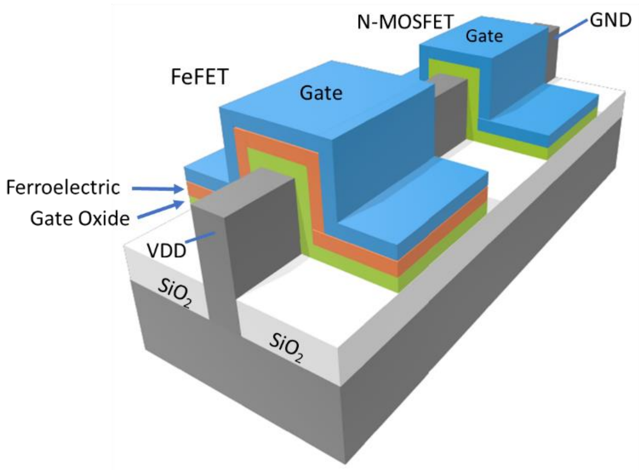

Figure S1. FeFET and MOSFET in FeFET spiking neuron circuits. FeFET has an extra ferroelectric layer between the gate metal and oxide.

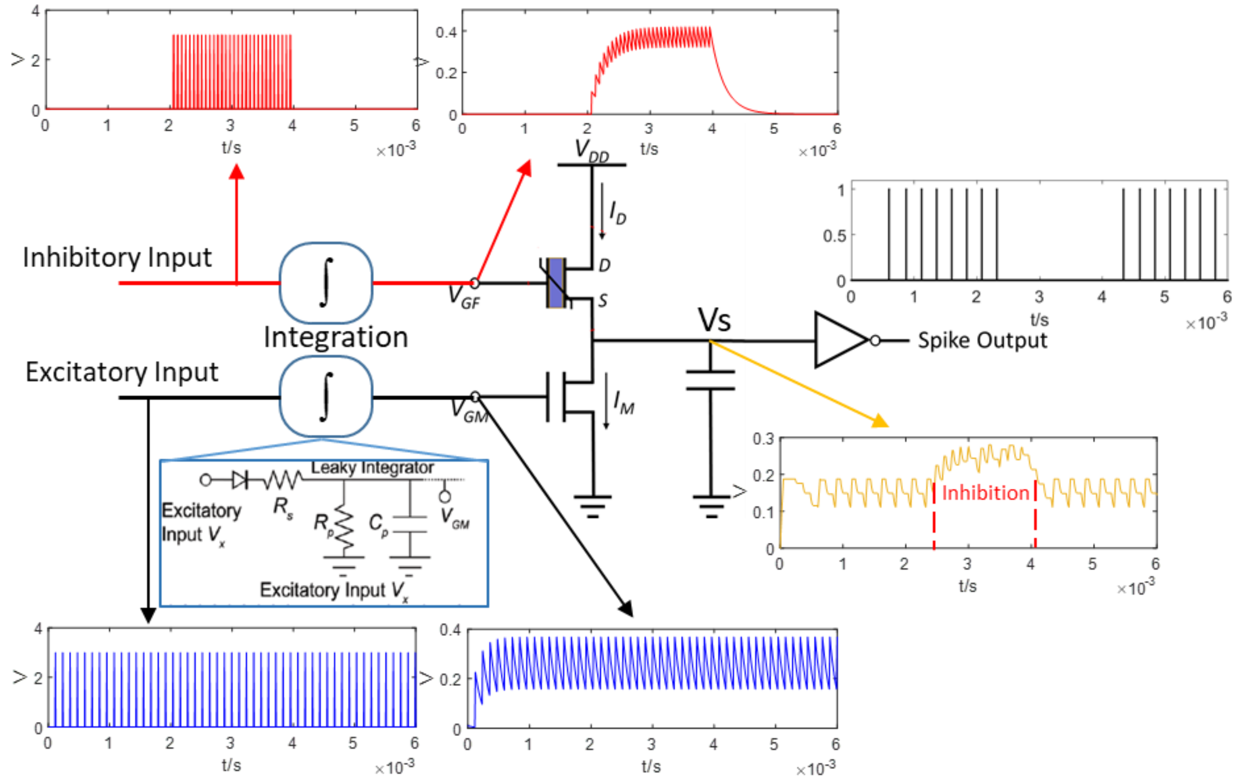

Figure S2. FeFET spiking neuron with Leaky Integrators that integrates the spike trains into input voltage of Vgm and Vgf. Such as design enables the frequency tuning through presynaptic spikes. The input signals are rectangular waveforms that can be either event-based or digital. Note that the oscillation output of V_S is tuned by the frequency of excitatory input, which controls the steps of discharging of capacitor C, while the inhibition is achieved by the integration of inhibitory input spikes.

To demonstrate that our model matches with experiments, we fit our model to the experimental data in (Wang 2018) and show an example of the validation based on the condition that V_{DD}=3.3V, V_{GF}=0.8V, C=0.1uF and V_{GM}=1.3V. Figure S3 plots the oscillation waveform of V_S and I_D - V_S curves of hysteresis loops from experimental data in (Wang 2018). Figure S4 shows the comparison between oscillation waveforms plotted from experimental data (blue) in (Wang 2018) and simulation results with dynamical behavior model (red).

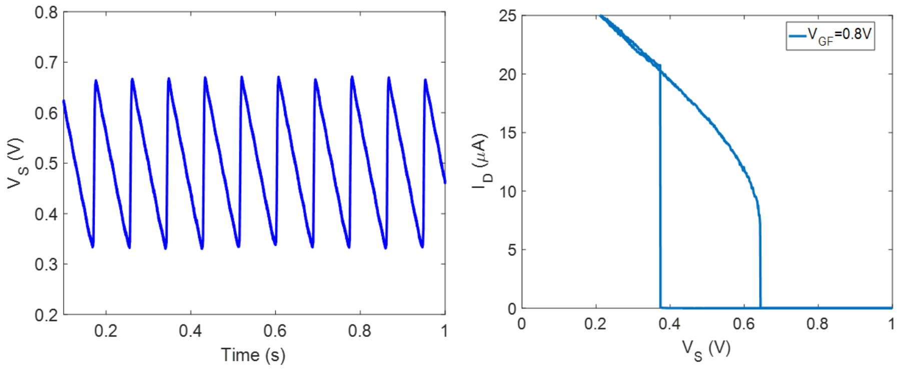

Figure S3 The waveform of V_S and I_D - V_S curves of hysteresis loops from experimental data in (Wang 2018) .

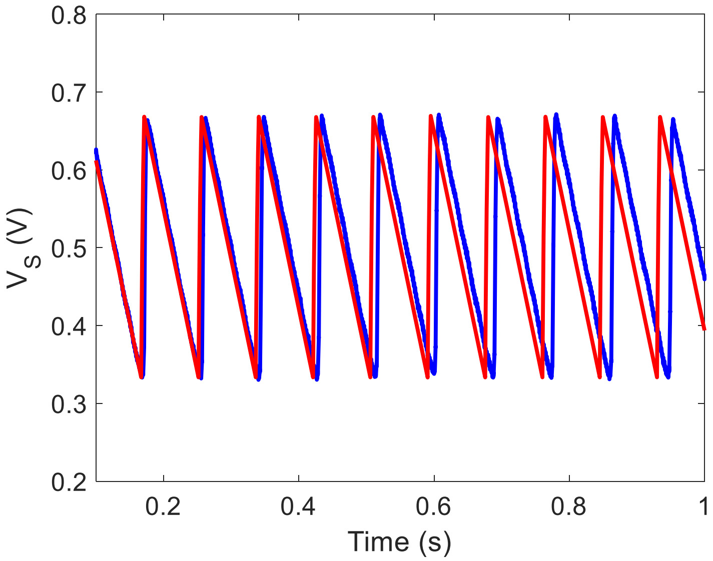

Figure S4. Plot of oscillation with experimental data (blue) and simulation results with dynamical behavior model (red)
